# Photo-ignition process of multiwall carbon nanotubes and ferrocene by continuous wave Xe lamp illumination

**DOI:** 10.3762/bjnano.8.14

**Published:** 2017-01-13

**Authors:** Paolo Visconti, Patrizio Primiceri, Daniele Longo, Luciano Strafella, Paolo Carlucci, Mauro Lomascolo, Arianna Cretì, Giuseppe Mele

**Affiliations:** 1 Department of Innovation Engineering, University of Salento, Lecce 73100, Italy; 2 Institute for Microelectronics and Microsystems - IMM-CNR, Department of Lecce, University Campus, Lecce 73100, Italy

**Keywords:** absorption spectra, CW Xe light source, metal nanoparticle ignitors, multiwalled carbon nanotubes, photo-induced ignition

## Abstract

This work aims to investigate and characterize the photo-ignition phenomenon of MWCNT/ferrocene mixtures by using a continuous wave (CW) xenon (Xe) light source, in order to find the power ignition threshold by employing a different type of light source as was used in previous research (i.e., pulsed Xe lamp). The experimental photo-ignition tests were carried out by varying the weight ratio of the used mixtures, luminous power, and wavelength range of the incident Xe light by using selective optical filters. For a better explanation of the photo-induced ignition process, the absorption spectra of MWCNT/ferrocene mixtures and ferrocene only were obtained. The experimental results show that the luminous power (related to the entire spectrum of the Xe lamp) needed to trigger the ignition of MWCNT/ferrocene mixtures decreases with increasing metal nanoparticles content according to previously published results when using a different type of light source (i.e., pulsed vs CW Xe light source). Furthermore, less light power is required to trigger photo-ignition when moving towards the ultraviolet (UV) region. This is in agreement with the measured absorption spectra, which present higher absorption values in the UV–vis region for both MWCNT/ferrocene mixtures and ferrocene only diluted in toluene. Finally, a chemo-physical interpretation of the ignition phenomenon is proposed whereby ferrocene photo-excitation, due to photon absorption, produces ferrocene itself in its excited form and is thus capable of promoting electron transfer to MWCNTs. In this way, the resulting radical species, FeCp2**^+∙^** and MWCNT^−^, easily react with oxygen giving rise to the ignition of MWCNT/ferrocene samples.

## Introduction

The photo-ignition process of carbon nanotubes (CNTs) was observed for the first time accidentally by exposing single-wall carbon nanotubes (SWCNTs) to the flash of an ordinary camera [1]. Following this, studies [2] highlighted that this photo-effect occurs in air for different types of SWCNTs prepared with different methodologies and weight percent of CNTs (in the range 50–90 wt %) with respect to the Fe metal catalyst mixed with them. The authors conjectured that the ignition and combustion occur when there is a local increase in temperature sufficient to initiate the oxidation of carbon. Their interpretation was that SWCNTs lend themselves to this photo-effect due to their black color, which allows better absorbing of the visible flash light and transmits the resulting thermal energy to the Fe nanoparticles.

The local transient high temperature inside the nanotubes must be at least ≈1500 °C in order for a structural reconstruction process to take place. This results in a permanent change in the SWCNT structure to a non-tubular structure, rather than merely elastically deforming. Furthermore, this reconstruction also happens after flash exposure even if ignition and combustion do not occur. Previous research work has provided the following results: the average light power required to ignite SWCNTs with density of 0.2 g/cm^3^ is 100 mW/cm^2^, while for high density, compact SWCNTs (>1 g/cm^3^), an average light power of 300 mW/cm^2^ was required [1–2].

Braidy et al. [3] focused their studies on the analysis of the post-ignition of the sample, after exposing the SWCNTs to the camera flash. By means of X-ray diffraction analysis of the residual dust, they found that this solid material is mainly composed of Fe_2_O_3_, with traces of Fe_3_O_4_, resulting from the Fe catalyst. A TEM investigation was also performed revealing that this material is composed of two different morphologies of oxides: small nanoparticles trapped within a network of residual SWCNT bundles and large randomly interconnected or fused grains. This implies that temperatures higher than 1500 °C were reached in localized points within the raw SWCNTs, confirming the hypothesis put forward in [1] and [2].

Further experiments were carried out to determine the cause of the photo-induced ignition in the SWCNTs [4]. In these tests, SWCNTs (synthesized by the high-pressure carbon monoxide process from Carbon Nanotechnologies, Inc., which utilizes Fe as a growth catalyst for the SWCNTs) and Fe powder are ignited if exposed to the camera flash. Under the same conditions, the purified SWCNTs showed no reaction. The authors postulated the following theory: photons emitted by the camera flash are absorbed by catalytic metal nanoparticles causing very high temperatures inside the sample; this occurs because Fe is less conductive than SWCNTs. In fact, they suggested that the heat is dissipated mostly in the CNT bundles with their high conductivity and interconnection, whereas the Fe nanoparticles are comparatively better as insulating heat. Therefore, the Fe particles can store enough thermal energy to reach the right temperature to oxidize. The authors supposed that the SWCNTs provided a stabilizing support to the Fe particles, avoiding their spontaneous ignition until they are exposed to a suitable energetic stimulus (such as light energy of the camera flash). Finally, according to the authors, the pyrophoric nature of Fe nanoparticles present within CNT bundles is the main reason the SWCNTs flash ignite, rather than any other physical/chemical characteristic of the MWCNTs. The results reported in [4] for SWCNTs have been confirmed also for multiwall carbon nanotubes (MWCNTs) in [5]. In fact, in this research work, the MWCNTs showed the same stabilizing behavior as the metal particles dispersed within them.

Assuming the role of metallic additives dispersed in the CNTs is pivitol [1–2,4], Sysoev et al. [6] gave a qualitative description of the processes that occur during photo-ignition of carbon nanotubes. This analysis determined the roles and functionalities of the different stages of combustion. At the first stage (during flash ignition), the oxidation of metal impurities dispersed in the CNT bundles takes place. At the next stage, CNT heating by chemical reaction occurs. This stage lasts 1.2 ms, according to [1], and finishes with an explosion. The explosion appears to occur as a consequence of the combustion sources arising in separate segments initiating a combustion wave. Tseng et al. [7] showed that the amount of catalyst (Fe particles embedded in CNTs) plays a key role for ignition, where a higher content of catalyst allows for easier ignition.

The main features of the SWCNT photo-ignition process (with 50% Fe content) using a camera flash and utilizing light sources with two different pulse time durations were studied in [8]. In addition, selected wavelength ranges of the incident light beam were suitably obtained by using optical filters. The study highlighted that, regardless which of the optical filters were used, the minimum energy needed for the ignition trigger only depends on the light pulse duration. With a pulse duration of 0.1 ms, an energy of about 30–35 mJ for each pulse is required to trigger the ignition of a loosely compact SWCNT sample mixed with Fe, whereas with a pulse duration of 9 ms, about 80–90 mJ/pulse were needed to achieve the same results. The authors also investigated on the minimum energy to ignite samples with various concentrations of Fe, using a pulse time duration of 9 ms. The reported results showed that a higher concentration of Fe reduces the minimum energy needed to trigger ignition [8–9]. The same results were also confirmed by other published research works [10–11].

**Figure 1:**
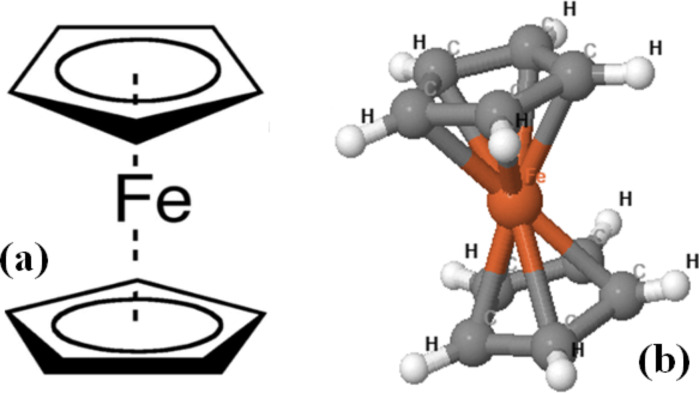
Ferrocene molecular structure (a) and its view as a 3D model (b).

The use of nanostructured materials as a means for distributed ignition and combustion improvement in propulsion applications (e.g., homogeneous-charged compression ignition (HCCI) engines, liquid rocket fuel sprays and enhanced flame stabilization in gas turbine engines) was patented in 2009 [12]. Since then, the properties of nanostructured materials as ignition agents have been studied [10,13–15]. In these works, the combustion ignition agent was constituted of the combination of metallic nanoparticles with CNTs mixed with different fuel mixtures (i.e., hexane/acetone, ethylene/air). On the other hand, ferrocene (FeCp_2_), whose molecular structure is shown in Figure 1, was the first pure hydrocarbon derived from iron and was accidentally discovered in 1951 [16]. Starting from its discovery, many other chemical compounds derived from it were synthesized and utilized in different research areas. The chemistry of ferrocene and its derivatives is actually well known as well as their chemical/physical features, which has proven very useful for different technical applications. In fact, nowadays, ferrocene and its derivatives mixed with other gaseous or liquid materials find more and more use in several areas [17] such as asymmetric catalysis, nonlinear optics [18], electro-chemistry [19] (due to the quasi-reversible oxidation of iron, e.g., Fe(II) oxide), and finally, in advanced fuel combustion processes.

Ferrocene photochemistry has been analyzed in detail [20–24]. Ferrocene is usually quite stable under visible irradiation, however, chemical modification may occur in the presence of light, or ferrocene may be used as an excited state quencher or photo-sensitizer (i.e., for the catalysis of photo-chemically induced reactions). From a fundamental viewpoint, the photo-chemical behavior of ferrocene and its derivatives is a topic of increasing interest. New applications are emerging in which ferrocene acts as a redox center to allow the occurrence of energy and electron transfer processes.

This innovative research field was recently developed in [25–26]. In more detail, the photo-induced ignition of MWCNTs containing ferrocene metal nanoparticles by using a Xe flash lamp was used to photo-ignite gaseous methane/air mixtures. This resulted in a more rapid and homogeneous combustion compared to ignition triggered by a traditional spark plug. In their work, the authors used 20 mg samples of MWCNTs containing 75 wt % ferrocene as ignition agents, and then added this to the methane/air gaseous mixture. The experimental results showed that the photo-induced ignition of a gaseous fuel mixture triggered by MWCNTs determines a higher combustion pressure gradient and a higher peak pressure with respect to spark-induced ignition for all the tested methane/air ratios. In addition, the high-speed camera images showed that the light-induced ignition using MWCNT/ferrocene mixtures as ignition agents leads to a more distributed homogeneous-like combustion, and therefore, to a faster consumption of the gaseous fuel mixtures without the formation of a discernible flame front.

In all reported research works, a pulsed flash lamp has always been used to trigger the ignition of the CNTs enriched with metal impurities. To our knowledge, a continuous wave (CW) Xe lamp has never been employed for this purpose, thus no investigation into the photo-ignition phenomenon has been conducted using this type of light source.

The aim of this research work is (i) to investigate and characterize the photo-ignition phenomenon of the MWCNT/ferrocene mixture by using CW Xe light source in order to better clarify the photo-induced ignition from a chemico-physical point of view; and (ii) to evaluate the power/energy ignition thresholds by using a different type of light source compared to that used in the previously mentioned works.

The experimental photo-ignition tests were carried out using varying weight ratios of mixtures, incident luminous power, and wavelength range of incident Xe light. In addition, in order to better understand the photo-induced ignition process, absorption spectra measurements of MWCNT/ferrocene mixtures and ferrocene were carried out after proper sample preparation [28].

## Experimental

The preparation procedure for obtaining reliable absorption spectra of the nanostructured material using a UV–vis spectrophotometer will be described. Furthermore, the experimental setup used to characterize the ignition of the MWCNT/ferrocene mixtures, the variable parameters such as MWCNT/ferrocene weight ratios, as well as the luminous power and wavelength range of the CW Xe incident beam will be presented.

### Material preparation method and absorption spectra

**Figure 2:**
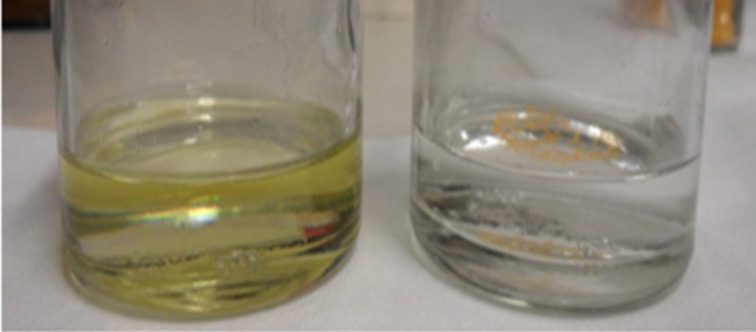
Ferrocene diluted in toluene (left) and ferrocene diluted in water (right) after sonication operation.

The absorption spectra of ferrocene and the MWCNT/ferrocene mixture were determined for comparison purposes. Concerning the measurements on ferrocene, toluene was selected as solvent because in order to obtain a homogeneous liquid solution, toluene appeared a better solvent compared to water. In Figure 2, water and toluene are shown after adding ferrocene followed by sonication. In the glass on the left containing the ferrocene/toluene solution, the sample appears more homogeneous than in the glass on the right containing the ferrocene/water solution.

The sonication operation, carried out employing the ultrasonic sonicator Bandelin SONOREX™ SUPER, was performed for a time duration variable from ten seconds up to five minutes on a solution of toluene (8 mL) and different amounts of ferrocene (as reported in Table 1). The sonication duration, although varied over a broad temporal range, did not highlight any significant changes in the homogeneity of the solution, even for higher ferrocene concentrations in toluene. In Table 1, the different amounts of ferrocene used in absorption spectra measurements are reported.

**Table 1:** Solvent type and amount of ferrocene used in this work.

Sample #ID	Solvent	Volume (mL)	Quantity of ferrocene (mg)	Volume ratio

#1	toluene	8	4.7	0.053%
#2	toluene	8	3.7	0.042%
#3	toluene	8	2.5	0.028%
#4	water	8	3.7	0.042%

**Figure 3:**
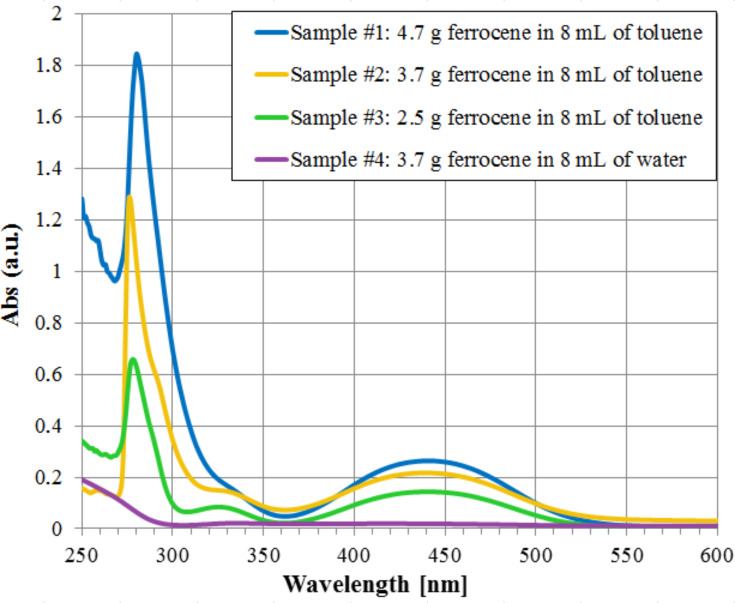
Absorption spectra of ferrocene diluited in toluene or water, varying the ferrocene concentration in 8 mL of solvent.

After sonication, the solution was introduced into the spectrophotometer (JASCO, V-660 UV-VIS) to obtain the absorption spectra, which was normalized with respect to toluene and is shown in Figure 3. For each concentration of ferrocene in toluene, the absorbance curves present a considerable peak at about 280 nm and a wider peak around 440 nm. Moreover, the absorbance values increase with the ferrocene concentration. The spectrum resulting from the ferrocene/water solution (also reported in Figure 3), normalized with respect to water, does not present significant peaks. This was expected because of the insolubility of ferrocene in water.

**Figure 4:**
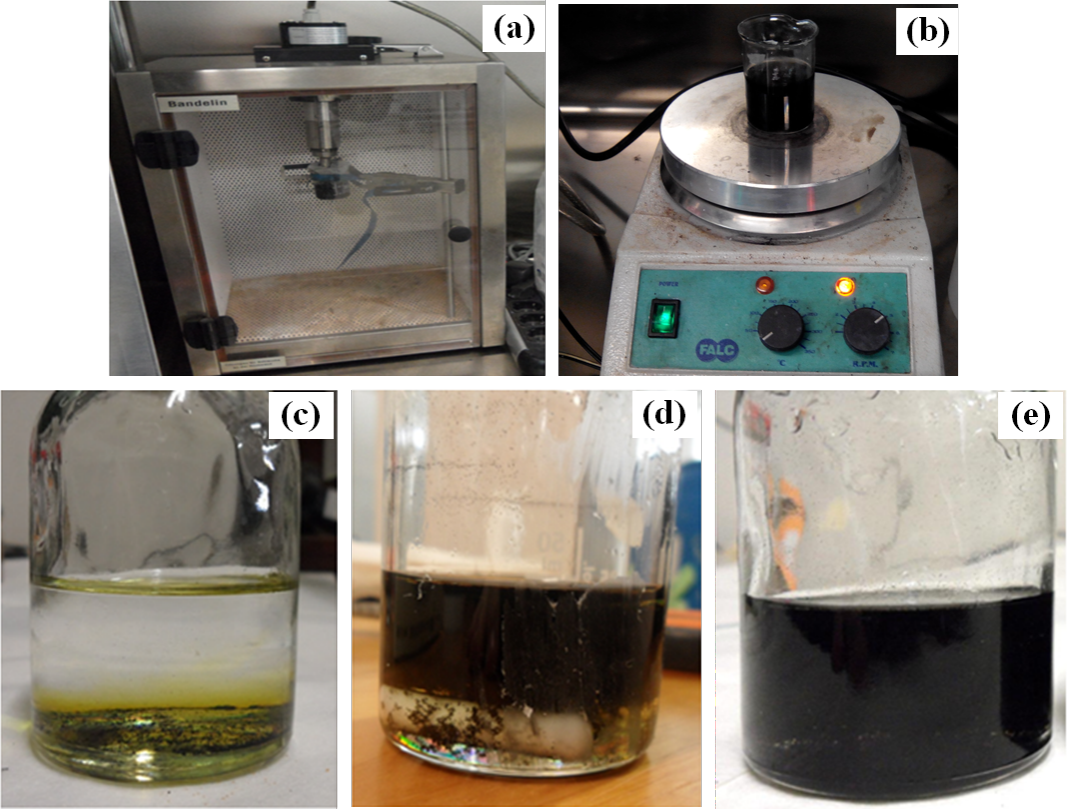
Image of sonicator probe Sonopuls Bandelin HD 2070 during the sonication treatment (a) and magnetic stirrer (FALC F70 model) to disperse the sample and obtain a more homogeneus solution (b). MWCNT/ferrocene mixture in toluene, before the treatment (c), after ten minutes of sonication and other ten minutes of magnetic stirring (d) and after another sonication for ten minutes (e).

To obtain the absorption spectra of the MWCNT/ferrocene mixture, the following procedure was adopted. Initially a material with weight of 9.5 mg (mixture of MWCNTs with 75% ferrocene by weight) in 25 mL of toluene (equal to a volume ratio of 0.029%) was used. To obtain a homogeneous solution, an ultrasonic probe (Figure 4a) and a magnetic stirrer (Figure 4b), were employed, each for ten minutes. The result was a visible change in the solution from that shown in Figure 4c to Figure 4d. The solution presented high opacity, large agglomerates and very fast precipitation of solids. These problems were not resolved by subjecting the sample to an additional ten minutes of sonication (Figure 4e).

**Figure 5:**
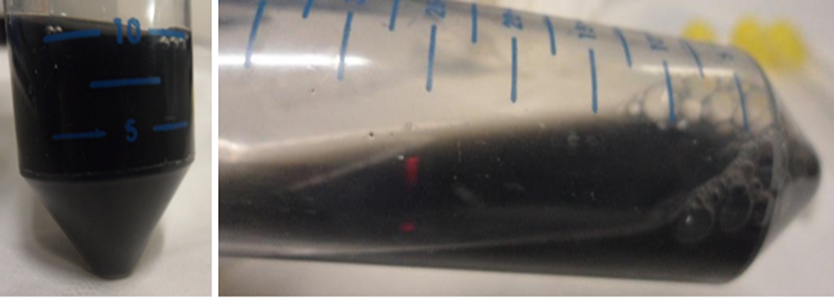
MWCNT/ferrocene mixture after adding SDS surfactant.

Employing sodium dodecyl sulfate (SDS), the dispersion of the mixture in the solvent was facilitated [28]. Using 250 mg of SDS in 25 mL of water, equal to a volume ratio of 1%, the ultrasonic probe and the magnetic stirrer were applied for ten minutes. The solution then appeared more homogeneous (Figure 5) than the previous ones, allowing for a reliable measurement of the absorption spectrum of the mixture.

**Figure 6:**
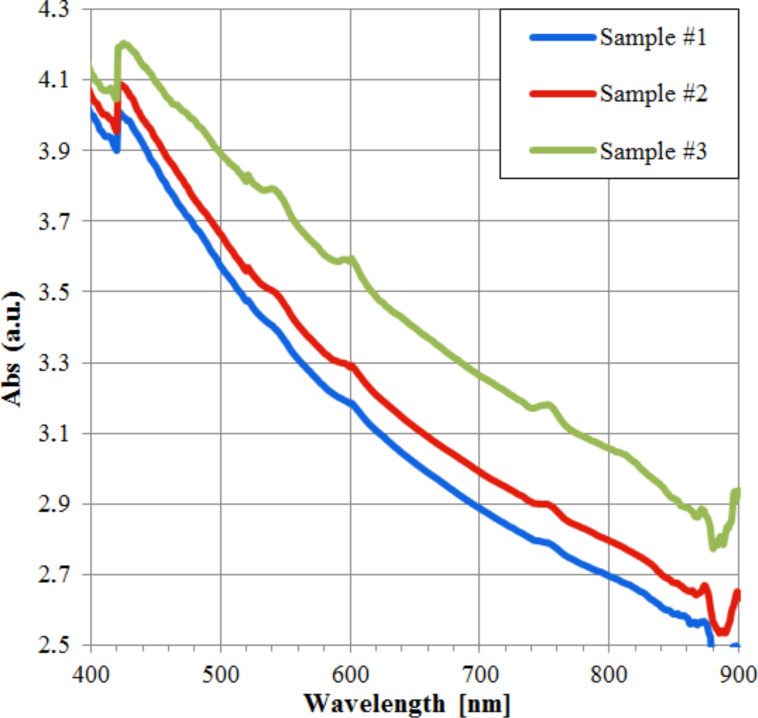
Absorption spectra of MWCNT/ferrocene soon after sonication and magnetic stirring (Sample 1), after one hour (Sample 2) and after a further sonication (Sample 3).

Three absorption tests were performed on the same solution: the first test was carried out soon after the sonication treatment (ten minutes) and magnetic stirring (ten minutes), providing, as result, the Sample 1 curve shown in Figure 6. After one hour, the same solution was again inserted into the spectrophotometer obtaining the red curve (Sample 2). Subsequently, after a further sonication for ten minutes, a new measure was made obtaining the green curve (Sample 3). The achieved results show that the absorption of the MWCNT/ferrocene mixture increases going from the visible toward ultraviolet region, as reported in the literature [15,27–28].

### Experimental setup for photo-ignition tests

**Figure 7:**
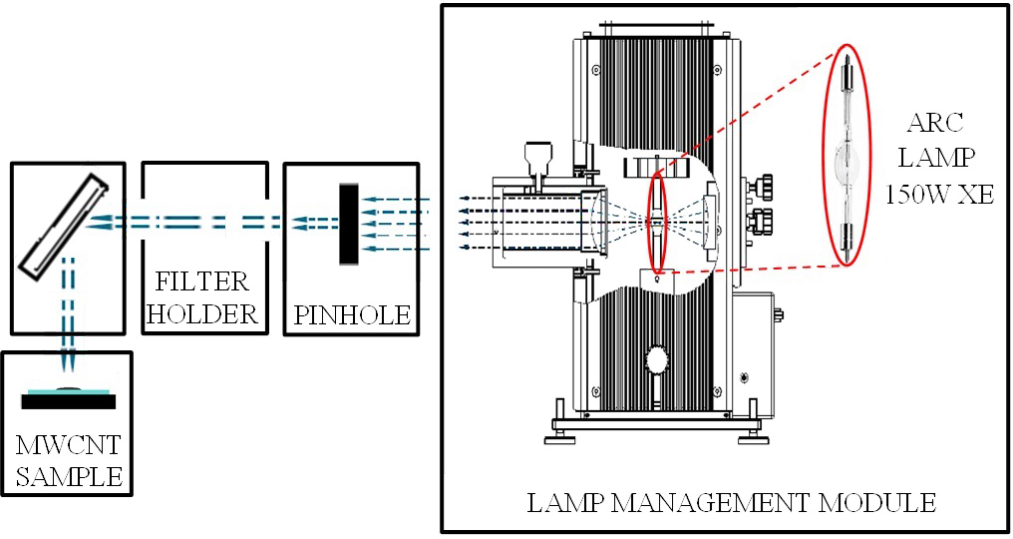
Schematic of the experimental setup for photo-ignition tests of the MWCNT/ferrocene mixture using a CW Xe lamp.

**Figure 8:**
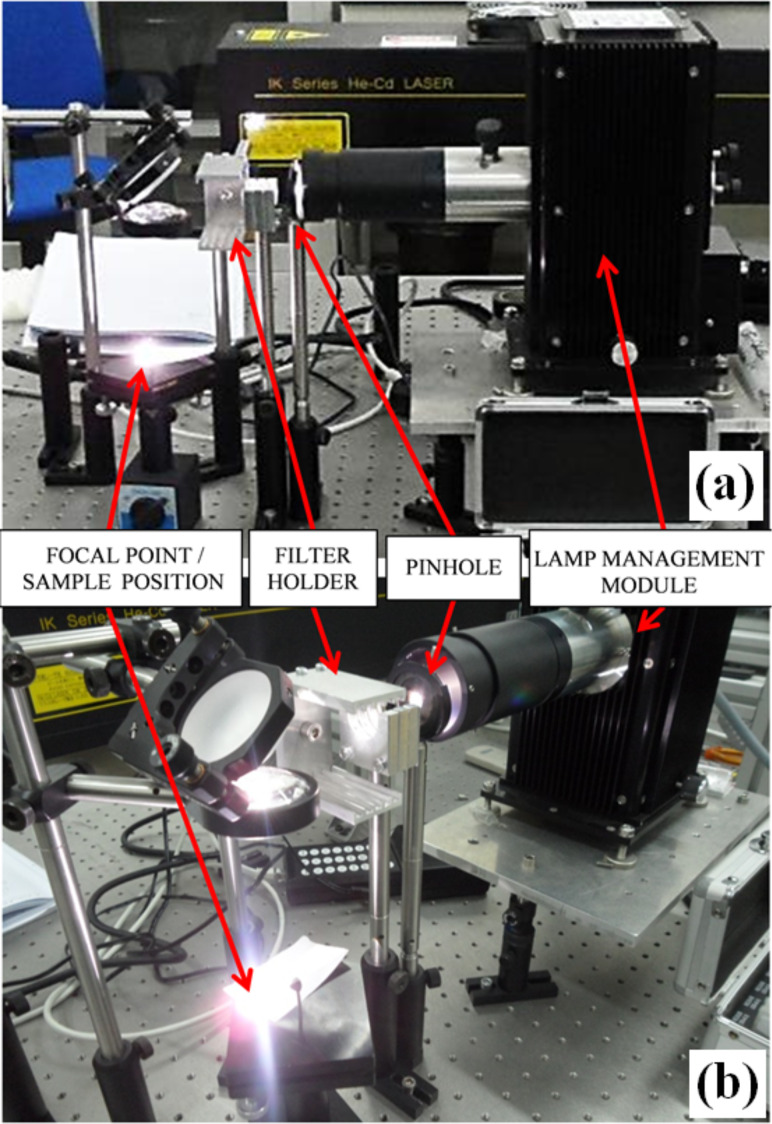
Images of the experimental setup for photo-ignition tests: lateral view (a) and front view (b).

**Figure 9:**
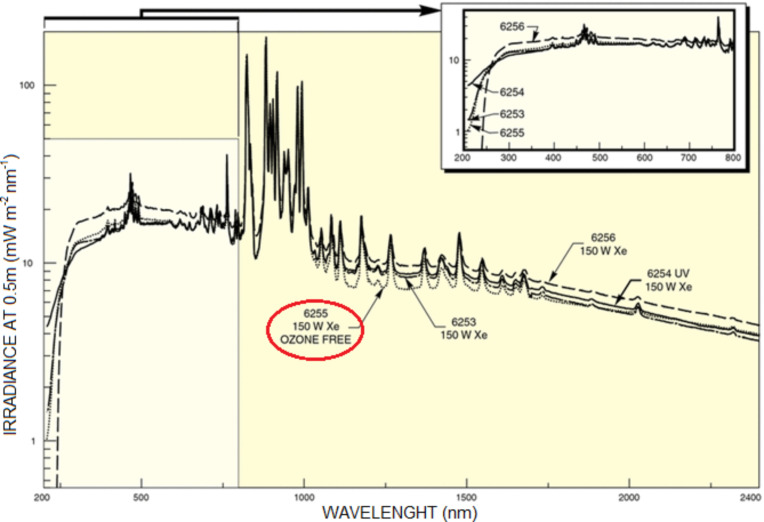
Typical spectra of a Xe arc lamp. The emission spectrum of the 150 W LSB521 Ozone-free Xe light source used in the experimental setup is highlighted.

A sketch of the experimental setup is shown in Figure 7. Using this setup, the ignition tests of MWCNT/ferrocene mixtures, subjected to a continuous wave light source, were carried out. The adopted CW Xe lamp is highlighted in red in the “lamp management module” block. Then, the generated light was reduced with a pinhole, properly filtered, and focused with suitable lenses for igniting the MWCNT sample placed in the holder. In the experimental setup used to perform the ignition tests (Figure 7 and Figure 8), the MWCNT/ferrocene sample was located in a position indicated in Figure 8 as “focal point/sample position”, at which the Xe light beam presents an almost uniform, circular illumination area, having a diameter of about 9 mm (illumination area, *S* = 63.6 mm^2^). The Xe lamp, whose emission spectrum is shown in Figure 9, is the LSB521 Ozone-free Arc lamps 150W Xe model from LOT-Quantum Design.

**Figure 10:**
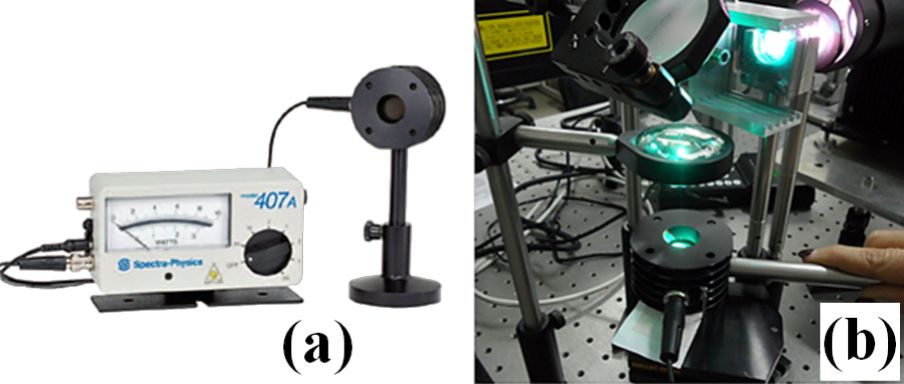
Laser power meter, Spectra Physics, Analog 407A (a) and view of the sensor positioned at the height of the focal point to measure the light spot power (b).

Along the optical path of the light beam generated by the lamp, a small circular opening (pinhole), which partitions the peripheral parts of the light radiation, was introduced. In this way, a more uniform luminous beam was obtained at the sample. The optical filters were arranged on proper supports (filter holder in Figure 8) placed after the pinhole. Two kinds of filters (Thorlabs) have been employed: neutral density (ND) filters, which attenuate the light radiation intensity of a given percentage factor evenly in the whole wavelength range covered by the lamp spectrum, and frequency selective filters, i.e., long pass (LP) filters and short pass (SP) filters, which attenuate certain wavelengths while others pass unchanged. Combining both LP and SP filters it was possible to select a specific wavelength range (bandpass filter) of the Xe light source. By means of a laser power meter (model 407A, shown in Figure 10a), the luminous power emitted by the CW Xe lamp was measured by placing the sensor at the focal point where the MWCNT/ferrocene mixture is located (Figure 10b).

The thermopile detector, employed in the measurements, is capable of measuring CW light power from a few mW up to more than 20 W and can withstand 20 kW/cm^2^ average CW power density. Readings are extremely precise because the absorbance of the detector varies only by ±1% in the range 400–1000 nm and by ±3% between 250 nm and 11 µm. Since the detector diameter is equal to 18 mm (with an area of 2.54 cm^2^), the whole illumination area of the light beam (≈0.64 cm^2^) falls within it (Figure 10b).

## Results

In the following, the results derived from the experimental measurements are illustrated. Initially, the minimum power thresholds necessary to trigger the ignition of the MWCNT/ferrocene mixtures will be reported for different mixture weight ratios and using the entire lamp spectrum. Subsequently, the light power threshold necessary for triggering the ignition when changing the wavelength range of the incident light on the sample will be presented and analyzed. Finally, a chemico-physical interpretation of the results will be presented by comparing with the results already reported in the literature.

### Ignition process with varying mixture composition and wavelength range

As reported in the literature, even if the CNTs are exposed to a light pulse with lower power than that required to trigger the photo-induced ignition, they are oxidized and undergo a total structural reconstruction in the presence of air or inert gases or in vacuum [1–2,7]. Therefore, each sample was illuminated only once during the experiment, regardless of whether the sample was ignited or not.

**Figure 11:**
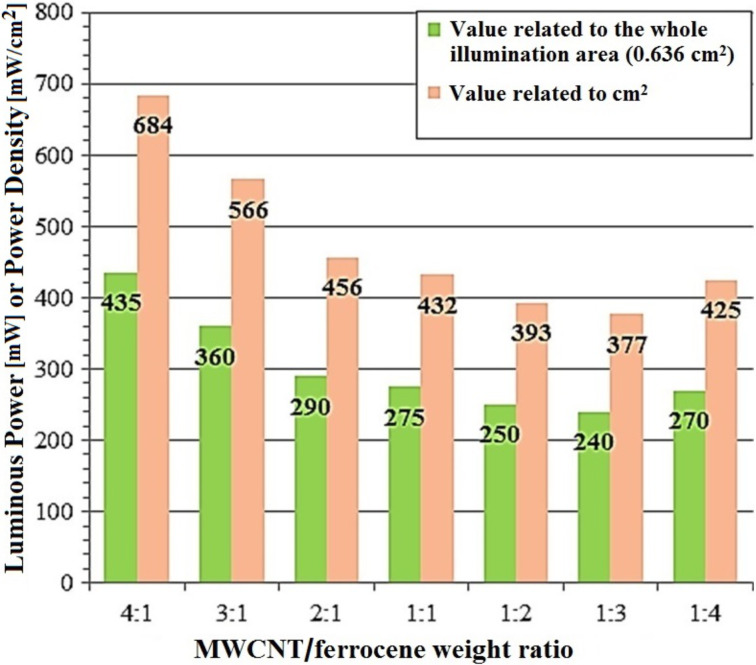
Light power thresholds for ignition of MWCNT/ferrocene mixtures found by varying the weight ratio.

The purpose of the tests was to search for the minimum power of light needed to trigger the ignition of the sample subjected to the luminous flux. Using the setup of Figure 8, the ignition tests were performed varying the weight ratio of MWCNT/ferrocene from 4:1 to 1:4, using the whole lamp emission spectrum (Figure 9) and making use of the optical attenuation ND filters (for adjusting to the beam power level that reaches the sample). The obtained results, reported in Figure 11, show that the 1:3 MWCNT/ferrocene mixture requires the least power to be ignited (240 mW which corresponds to a power density threshold of 377 mW/cm^2^). An uncertainty of about ±4% was estimated, relative to the reported threshold light power values.

As already reported in the literature, the luminous energy value needed to trigger the ignition of SWCNT/Fe decreases with increasing Fe [7–11]. In this work, even if a different type of light source (i.e., CW Xe lamp instead of pulsed Xe lamp) has been used, a similar dependence between the ferrocene concentration and the minimum power needed for ignition was observed. The best weight concentration of ferrocene was 75% with respect to CNTs (MWCNT/ferrocene ratio 1:3).

As a second experiment, a MWCNT/ferrocene mixture with a weight concentration of 1:3 was used, which was the mixture requiring the lowest ignition power. Making use of frequency selective optical filters (i.e., LP and/or SP filters), different wavelength ranges of light were selected to illuminate the sample. For each wavelength range, the luminous power threshold for ignition was found by using light attenuating ND filters.

**Figure 12:**
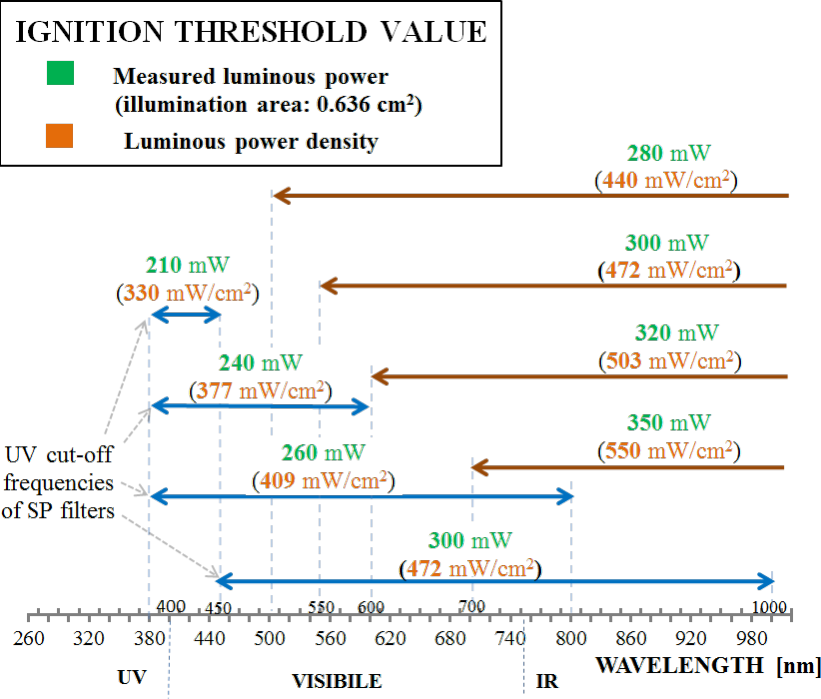
Light power thresholds for ignition using filters to select specific wavelenght ranges: low (blue arrows) and high pass filters (brown arrows).

In Figure 12, the luminous power thresholds obtained by employing SP filters (blue arrows) (each filter characterized by a UV cut-off frequency equal to about 380 nm) or LP filters (brown arrows) are reported. The measured power values related to the illumination area (0.636 cm^2^) are highlighted in green, while the calculated luminous power density values (mW/cm^2^) are shown in the brackets in orange.

**Figure 13:**
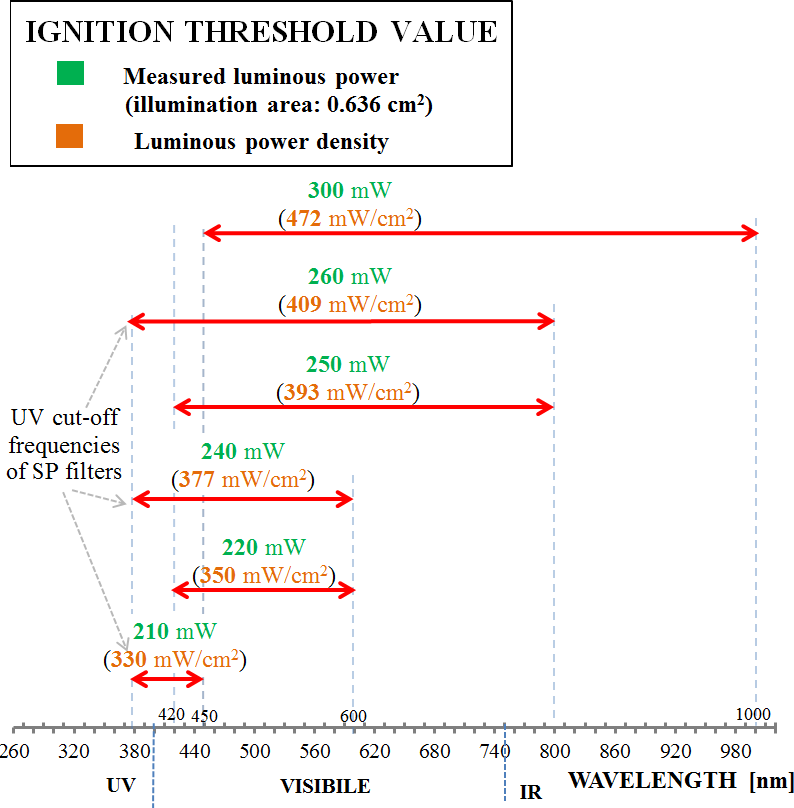
Light power thresholds for ignition using band pass filters.

In Figure 13, the luminous power thresholds and calculated power density values relative to the combined use of SP and LP filters (i.e., band pass filters) are reported. The results highlight that in order to ignite the MWCNT/ferrocene mixture, less power in the range 210–240 mW (namely 330–377 mW/cm^2^) is needed in the UV region.

**Figure 14:**
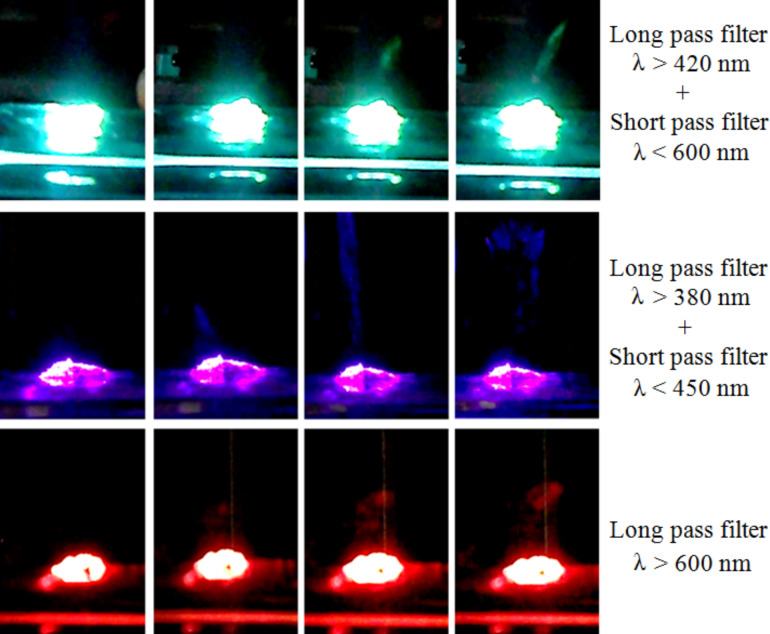
Frames extracted from videos recorded during ignition tests. The variable visible color of the incident light on the sample is due to the different wavelenght ranges selected by the optical filters. For each test, ignition was observed.

These results are consistent with the obtained absorption spectra (shown in Figure 3 and Figure 6). In fact, the absorption curves of ferrocene present a peak in the range 270–320 nm (however, this is not usable in the ignition tests of Figure 12 and Figure 13 because of the SP filter absorption in UV region) and a less intense but wider peak around 440 nm. Moreover, a higher absorption has been found for MWCNT/ferrocene mixtures illuminated by visible radiation with decreasing the wavelength up to 400 nm. In Figure 14, some frames are shown extrapolated from videos recorded during the ignition tests.

**Figure 15:**
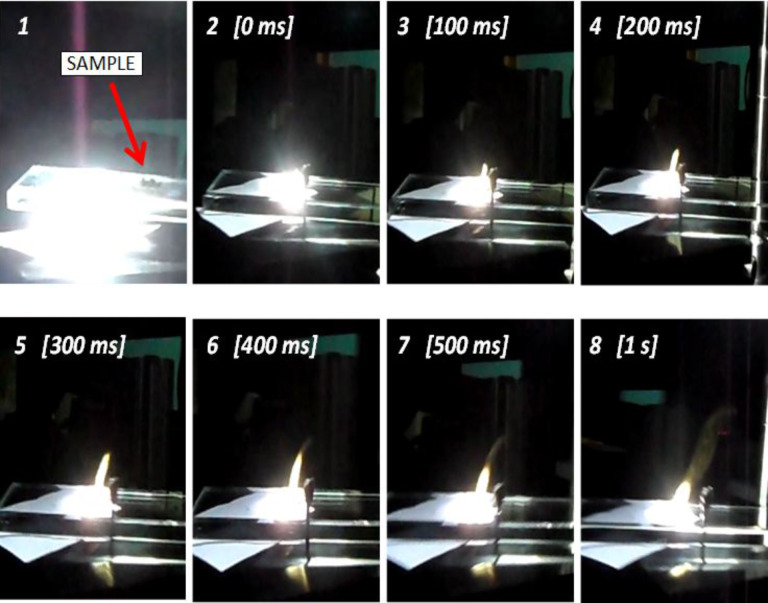
Captured pictures of the combustion process where the MWCNT/ferrocene sample is ignited by illumination of the whole spectrum of the Xe lamp. The first frame refers to the time just before the sample enters the white light beam path.

In the following pictures reported in Figure 15, the time evolution of the combustion process is shown when using the full spectrum of the Xe-lamp light beam, i.e., without any selective filters. The combustion flame is clearly visible (after only 100 ms, third frame) and it occurs for the 1:3 MWCNT/ferrocene mixture weight ratio, with a luminous power density equal or higher than 377 mW/cm^2^.

**Figure 16:**
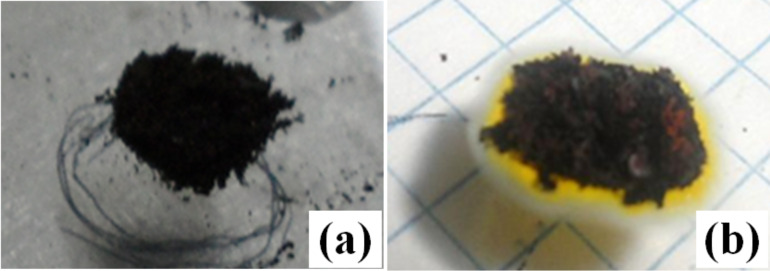
Sample images of MWCNT/ferrocene mixture (with weight ratio 1:3) positioned on the slide before (a) and after photo-induced ignition process (b).

In Figure 16, the images of MWCNT/ferrocene samples before and after the ignition process are shown. As reported in the literature, after ignition, the exposed samples display orange dots or clusters because of the presence of ferrocene in the mixture (more evident with increasing ferrocene concentration), indicative of iron oxide particles [9,15].

## Discussion

### A chemico-physical interpretation of obtained results

One of the central aspects in CNT chemistry and physics is their interaction via electron transfer. The intermolecular interactions with electronic charge transfers between nanotubes and ferrocene showed that this composite material can be used for converting solar energy into energy to promote an electron transfer by means of a photo-excitation process.

**Figure 17:**
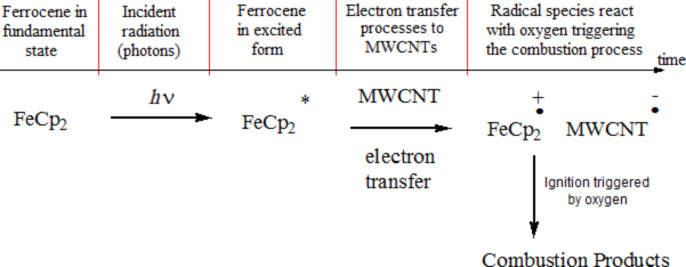
Chemical reactions relative to photo-induced electron and energy transfer in MWNTs–FeCp_2_ nanocomposites occurring during the photo-induced ignition process.

The chemical physical feature of the multiwalled carbon nanotube/ferrocene nanocomposites (MWCNT/FeCp_2_) responsible for starting the ignition process could be ascribed to the charge-separated state, MWCNT **^.^**^−^–FeCp_2 _**^.^**^+^, as expected by photo-induced electron transfer in the MWCNT–FeCp_2_ nanomaterial hybrid. This leads us to postulate intermolecular electron transfer to give MWCNT**^.^**^−^–FeCp_2_**^.^**^+^, depending on the selected wavelength range of the incident Xe light used to trigger the photo-induced ignition process. From a chemico-physical point of view, the suggested evolution of ignition process has been summarized in the Figure 17.

As reported in Figure 17, the photo-induced process of charge separation, promoted by UV–vis–IR light irradiation (photon energy *h*∙ν = *h*∙*c*/λ with 380 nm < λ < 1000 nm, *c* speed of light and *h* Planck constant), represents the key step of the MWCNT/Fe ignition process. In this scenario, as already widely reported in the literature, the presence of metal nanoparticles (in this work FeCp_2_) allows for the photo-ignited reaction.

In particular, the photo-excitation of FeCp_2_ produces ferrocene in its excited form FeCp_2_**^.^** which is capable of promoting electron transfer processes to MWCNTs. The radical species produced in this way, FeCp_2_**^.+^** and MWCNT**^.−^**, easily react with oxygen (O_2_) giving rise to a typical combustion reaction. Of course, the opportune combination of the proper incident light wavelength range and luminous power (as reported in Figure 12 and Figure 13) are important in order to ensure the success of the MWCNT/ferrocene ignition process.

The importance of the presence of oxygen for ignition was already observed in [2] where the authors reported that when the sample is more dense, then higher power is required to ignite the SWCNT material. They believe that the lack of oxygen access and the loss of heat into the bulk of the denser samples make their ignition more difficult. In more detail, if the CNT material is more dense, many bundles are in contact resulting in fast heat dissipation into the bulk. In conclusion, lower light energy density is required for ignition when bundles are separated and surrounded by oxygen [2].

## Conclusion

In this work, a full analysis of the ignition process of MWCNT/ferrocene mixtures, photo-ignited by using a CW Xe light source, was carried out. Although a different type of Xe light source (i.e., CW instead of pulsed Xe light, as was used in previous research works) has been used, photo-induced ignition was similarly obtained. The absorption spectra of MWCNTs/ferrocene mixtures revealed higher absorbance values moving from the visible toward UV region while with only ferrocene, a significant peak in UV region at 280 nm and a slight peak around 440 nm were obtained. The luminous power thresholds for triggering ignition were found for different mixture weight ratios varying the parameters of the CW Xe light incident on the sample such as the luminous power and the wavelength range. In order to select different wavelength ranges and to reduce the light source power, selective optical filters (LP and/or SP) and ND attenuating filters were employed. The results obtained by irradiating the sample with the full Xe lamp spectrum show that the MWCNT/ferrocene mixture with 1:3 weight ratio (i.e., 75% ferrocene by weight) requires the least luminous power to be ignited. Utilizing this weight ratio and selecting specific wavelength ranges with selective filters, we found that photo-induced ignition is obtained with lower luminous power in the 380–450 nm UV–vis range; this was in agreement with the obtained absorption spectra. Finally, a chemico-physical interpretation was given to explain the ignition phenomenon. The irradiating light on MWCNTs/FeCp2 samples produces ferrocene in its excited form capable of promoting electron transfer processes to MWCNTs. In this way, the formation of radical species, which easily react with oxygen, triggers the combustion process of the mixture.
